# Aerosol optical, microphysical, chemical and radiative properties of high aerosol load cases over the Arctic based on AERONET measurements

**DOI:** 10.1038/s41598-018-27744-z

**Published:** 2018-06-20

**Authors:** Yisong Xie, Zhengqiang Li, Li Li, Richard Wagener, Ihab Abboud, Kaitao Li, Donghui Li, Ying Zhang, Xingfeng Chen, Hua Xu

**Affiliations:** 1 Environment Protection Key Laboratory of Satellite Remote Sensing, Institute of Remote Sensing and Digital Earth, Chinese Academy of Sciences, Beijing, 100101 China; 2 Environmental & Climate Sciences Department, Brookhaven National Laboratory, Upton, New York 11973 USA; 3 Measurement and Analysis Research Section, Environment and Climate Change Canada, Ontario, L0L1N0 Canada

## Abstract

Columnar mass concentrations of aerosol components over the Arctic are estimated using microphysical parameters derived from direct sun extinction and sky radiance measurements of Aerosol Robotic Network. Aerosol optical, microphysical, chemical and radiative properties show that Arctic aerosols are dominated by fine mode particles, especially for high aerosol load cases. The average aerosol optical depth (AOD) of the selected Arctic sites in the sampling period is approximately 0.08, with 75% composed of fine mode particles. The fine mode fraction mostly exceeds 0.9 when AOD greater than 0.4. The ammonium sulfate-like component (AS) contributes about 68% of total dry aerosol mass for high-AOD events. The estimated compositions and back trajectories show that the transported aerosol particles from biomass burning events have large amounts of black carbon (BC) and brown carbon, while those from pollution events are characterised by large AS fractions. The instantaneous radiative forcing at the top-of-atmosphere is higher for the more absorbing components, and varies greatly with surface albedo and solar zenith angle. A regression model of columnar composition and radiative forcing within the atmosphere (RF_ATM_) for Arctic aerosol is established, showing that BC dominates a positive RF_ATM_ with a high warming efficiency.

## Introduction

Aerosol has important impacts on global climate change and atmospheric environment. However, there is still a lack of understanding of atmospheric aerosol, and as a result, aerosol radiative effect is one of the most significant sources of uncertainty in climate change assessment^1,2^. Particles transported from mid-latitude regions in Europe, North America and Asia^3,4^ episodically reach the Arctic region^5^, and significantly influence the radiative energy balance in the Arctic^6^. For example, carbonaceous particles can absorb solar radiation and heat the atmosphere, resulting in positive radiative forcing. On the other hand, black carbon particles deposited on snow and ice surfaces lead to a decrease in surface albedo and an increase in melting^7,8^.

Atmospheric models are able to obtain aerosol radiative properties in a global scale, but may have trouble providing accurate simulations, and have noticeable differences from the observations over specific regions (e.g., the Arctic region^3,9^). Thus, direct measurements are necessary for better simulations, especially for high aerosol load situations^10^. The Aerosol Robotic Network^11^ (AERONET) includes several long-term observation sites in the Arctic region and provides an online archive of the many derived aerosol parameters. Such ground-based remote sensing observations like satellite observations derive column average aerosol properties in ambient conditions^12^. Previous studies analysed the optical properties of Arctic aerosol using AERONET products^13–15^, and some used AERONET data to validate models and satellite retrievals^10,16,17^. However, few studies focused on the chemical composition and microphysical properties, which are essential to the assessment of optical and radiative properties^18^. Aerosol composition is one of the main sources of uncertainty in estimating the aerosol radiative effect^19^. Particularly in the Arctic region, light-absorbing aerosol components have significant impacts on the radiative effect because of the high surface albedo of ice and snow surfaces^20^.

The columnar mass concentrations of the main aerosol components can be quantitatively estimated by ground-based remote sensing measurements. With the development of composition estimation algorithms^21–25^, a modified aerosol composition model covering major tropospheric aerosol species and a method for inferring these components with optical and microphysical parameters^12^ have been proposed. In this study, we employ this method to obtain the columnar aerosol composition over the Arctic region under high aerosol load conditions. The relation between the estimated components and direct aerosol radiative forcing within the entire atmosphere is established for the first time.

## Data and Methods

### Arctic observation sites and AERONET data

We select 8 AERONET sites located within the Arctic Circle, where AERONET level 2.0 optical, microphysical and radiative parameters are available: Barrow (71.3 °N, 156.7 °W, BAR) in Alaska, Resolute_Bay (74.7 °N, 94.9 °W, RES) on Cornwallis Island, PEARL (80.1 °N, 86.4 °W, PEA) in the Canadian Arctic, Thule (76.5 °N, 68.8 °W, THU) and Ittoqqortoormiit (70.5 °N, 22.0 °W, ITT) in Greenland, Hornsund (77.0 °N, 15.6 °E, HOR) in Svalbard, Sodankyla (67.4 °N, 26.6 °E, SOD) in Finland, and Tiksi (71.6 °N, 128.9 °E, TIK) in Russia. Aerosol particles over the Arctic are significantly affected by emissions from mid-latitude regions^3^. Some Arctic sites located further south (i.e., near the Arctic Circle) are close to major emission sources; for example, BAR and RES suffer from American biomass burning, SOD and HOR are affected by European pollution, and TIK is disturbed by Russian agricultural combustion. In contrast, some sites located in remote or high Arctic regions (e.g., ITT and PEA) are less impacted by transported particles.

We retrieve all the available Version 2 inversion data that pass the level 2 quality assurance criteria^26^ for these 8 sites from the AERONET website (https://aeronet.gsfc.nasa.gov/, accessed on July 20, 2016). The parameters used in this study are aerosol optical depth (AOD), fine mode fraction (FMF), real and imaginary parts of the complex refractive index (RRI and IRI) at four wavelengths (440, 675, 870 and 1020 nm), volume size distribution, spherical fraction and instantaneous radiative forcing at the top-of-atmosphere and bottom-of-atmosphere. The uncertainties of AOD and FMF are 0.02^11^ and 0.1^27^, respectively. The uncertainties are larger for the microphysical parameters: 0.04 for RRI and 50% for IRI, and 35–100% for each bin of the size distribution depending on the radius^28^. Note the level 2 refractive index data are only computed when the AOD (at 440 nm) is larger than 0.4 to assure a good enough signal for the inversion. Since the derivation of the aerosol component mass concentrations requires the RRI and IRI, they are also restricted to high aerosol load cases. These cases make up only a small fraction of the total observations and they clearly only represent aerosol properties under severely polluted conditions in the Arctic region. They occur more frequently at sites near major emissions (e.g., approximately 8% of all successful inversions at TIK) than at remote sites (e.g., less than 1% at ITT and THU).

### Estimation of columnar aerosol composition

Aerosol components are quantitatively estimated from aerosol light absorption, size and particulate shape parameters provided by AERONET products according to the intrinsic relations between microphysical and chemical properties. First, a columnar aerosol composition model that contains black carbon (BC), brown carbon (BrC, also known as light-absorbing organic carbon), mineral dust (DU), ammonium sulfate-like (AS), sea salt (SS) and aerosol water uptake (AW) is established, as well as the spectral RRI and IRI, the lognormal size distribution and the spherical fraction of each individual component. Note that the AS component refers to light-scattering and fine-mode aerosol particles, including inorganic salts (e.g., sulfate) and organic species. Then, the microphysical parameters of the aerosol mixture with respect to assumed component volume fractions are calculated based on Maxwell-Garnett internal mixing model^25^, which is employed for Arctic aerosol considering the ageing process of transported particles from mid-latitude regions. It should be noted that by solely relying on remote sensing measurements, it is difficult to evaluate the physical transformation of aerosol particles over the Arctic (e.g., hygroscopicity variation and water phase alteration) due to the low temperature, so we do not take these complicated situations into account in this study. At last, total residual between the modelled and observed parameters that are weighed by their uncertainties is calculated. Searching for the minimum total residual yields the best composition results based on a look-up table approach. Moreover, the inferred component volume fractions can be transformed into columnar mass concentrations using component densities. More details of the algorithm can be found in our previous study^12^.

Errors of the estimated components are mainly from uncertainties of the AERONET inputs^23,24^. We employ a method similar to the one introduced in the previous study^12^ to assess the errors (see Supplementary Table S1). The largest errors for individual components are: IRI at 870 nm causes an error of 0.5% for BC, IRI at 440 nm causes 8.4% for BrC, spherical fraction causes 13% for DU, fine-mode volume fraction causes 11% for SS, and spectral average of RRI causes 23% for AS and AW, respectively. We should clarify that these values indicate the theoretically maximum errors, i.e., the errors when the parameter uncertainties individually reach their peaks. The practical composition errors should be smaller, because for the quality assured level 2 AERONET data it is unlikely that the uncertainties of all input parameters reach high levels at the same time; using multiple parameters to derive aerosol composition could also avoid significantly biased components caused by one parameter with large uncertainty. Nevertheless, it is difficult to directly verify the estimated components because of insufficient validation data, so the accuracy of the inferred aerosol composition should not be overestimated. Since the estimated components from individual measurements might have errors that are too large for reliable analysis^24^, we only give the component mass concentrations or fractions as average values for a day, aerosol event, or Arctic site in this analysis.

### Direct aerosol radiative forcing

The instantaneous direct aerosol shortwave radiative forcing is defined in this study as the difference in net fluxes (downward flux minus upward flux) due to aerosol under cloud-free conditions at the top-of-atmosphere (RF_TOA_) and at the bottom-of-atmosphere (RF_BOA_):

1 $$R{F}_{TOA}=({F}_{TOA}^{a\downarrow }-{F}_{TOA}^{a\uparrow })-({F}_{TOA}^{c\downarrow }-{F}_{TOA}^{c\uparrow })$$

2 $$R{F}_{BOA}=({F}_{BOA}^{a\downarrow }-{F}_{BOA}^{a\uparrow })-({F}_{BOA}^{c\downarrow }-{F}_{BOA}^{c\uparrow })$$

where *F*_*TOA*_ and *F*_*BOA*_ are the fluxes at the TOA and BOA, the superscript *a* and *c* indicate situations with and without aerosol, respectively, and the arrows denote upward or downward flux. Note the downward flux at the TOA is the same either for aerosol absent or aerosol present conditions, and the upward flux at the BOA can be approximated by the downward flux and surface albedo (SA), the RF_TOA_ and RF_BOA_ can be also written as:

3 $$R{F}_{TOA}={F}_{TOA}^{c\uparrow }-{F}_{TOA}^{a\uparrow }$$

4 $$R{F}_{BOA}=({F}_{BOA}^{a\downarrow }-{F}_{BOA}^{c\downarrow })(1-SA)$$

AERONET radiative forcing products are calculated by broadband fluxes from 0.2 to 4.0 μm, which are computed from aerosol parameters including size distribution, spectral AOD, single scattering albedo and phase function by using the radiative transfer module integrated in the AERONET inversion^29,30^. The AERONET-defined RF_TOA_ has the form of Eq. (3) and we directly use AERONET RF_TOA_ data. However, the RF_BOA_ is defined by AERONET as the difference of downward fluxes with and without aerosol, so we take the upward fluxes at the BOA into consideration by multiplying AERONET RF_BOA_ with the term 1-*SA*, as shown by Eq. (4). The SA is the spectral average of the surface albedo calculated by the AERONET Version 2 algorithm at the four operational wavelengths, instead of the average of the SA values in the whole solar range. The uncertainty in RF_BOA_ introduced by the assumed SA has previously been estimated to be less than 10%^29^. Radiative forcing within the atmosphere (RF_ATM_) is calculated by RF_ATM_ = RF_TOA_ − RF_BOA_.

## Results and Discussion

### Aerosol optical and microphysical properties over the Arctic

**Figure 1:**
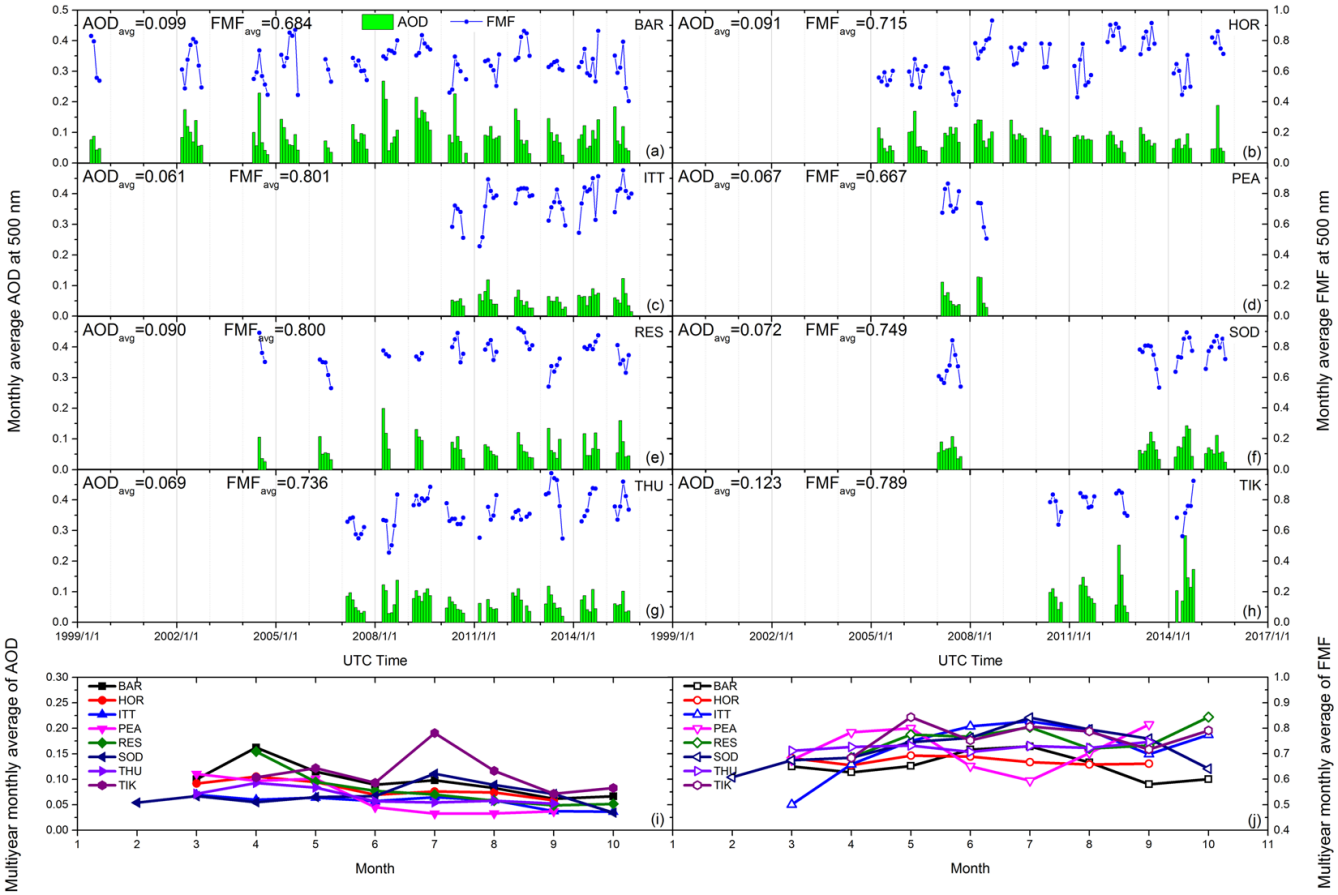
(**a**–**h**) Monthly average (avg) AOD and FMF at 500 nm for the Arctic sites; (**i**,**j**) multiyear monthly average AOD and FMF at all sites.

The average of all AOD at 500 nm in the sampling period at the Arctic AERONET sites is 0.08 ± 0.08. BAR and TIK have slightly higher AOD (0.10) due to emissions from North America and Russia, respectively. In contrast, the mean AOD at the remote ITT site is only 0.06, which represents the typical Arctic background value^16^. Seasonal AOD patterns for most of the sites in the Arctic, as shown in Fig. 1(i), are higher in spring (~0.10) and lower in summer and autumn (0.08 and 0.05, respectively), which is also reported by modelling results and chemical measurements^3,31^. The large AOD in spring might be associated with transported particles from boreal forest burning^10^ and pollution^32^, given that cyclonic activities in spring create opportunities for long-distance particle transport towards the Arctic in the free troposphere. The small AOD in summer-autumn, in contrast, is attributed to decreasing aerosol transport and increasing wet removal during this period^33,34^.

Fine mode aerosol particles dominate total extinction over the Arctic^35^, with an average FMF of 0.75 ± 0.16. Compared to other typical aerosol types^36^, the FMF of Arctic aerosol is more similar to urban/industrial or biomass burning aerosol rather than the low FMF for natural types (e.g., dust or oceanic aerosol). This likely indicates the significant impacts of anthropogenic or combustion sources (which mainly emit fine particles) on Arctic aerosol. The multiyear monthly averages of FMF shown in Fig. 1(j) illustrate that FMF over the Arctic is slightly low in spring (0.65 in March), which might be due to dust particles originating from Asia. Then, FMF increases in summer^15^ and reaches a high level of 0.77 in July (the opposite pattern at the PEA site may be due to the short data duration). From long-term observations (i.e., >5 years), we find that FMF during summer months at the HOR, ITT and THU sites shows generally increasing annual trends with moderate fluctuations, while for other sites or periods, there are barely notable trends.

**Figure 2:**
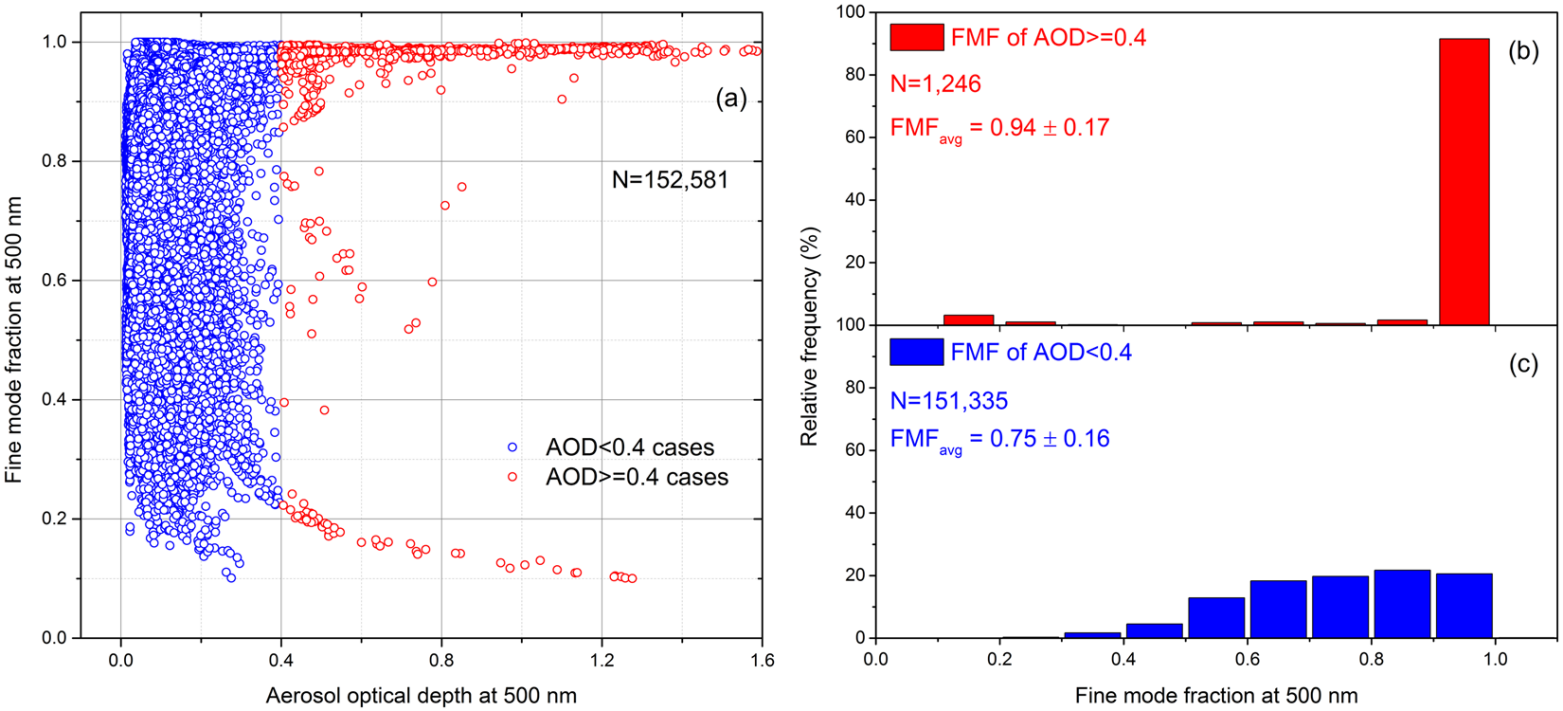
(**a**) FMF variations with AOD for all measurements at the Arctic sites. Probability distributions of FMF when (**b**) AOD > = 0.4 and (**c**) AOD < 0.4 are also shown.

Based on the FMF variations with AOD shown in Fig. 2, we notice that for small AOD cases (i.e., <0.4), FMF shows no clear dependence on AOD. In contrast, when AOD is larger than 0.4, about 91% of the data have a very large FMF (i.e., >0.90), indicating that most of the large AOD values are due to fine particles. Usually, the FMF can be interpreted as an index of anthropogenic sources when larger than 0.83^37^. The FMF dependency on AOD clearly reveals significant impacts of the transported fine mode particles on Arctic aerosol, especially under high aerosol load conditions. It is also noticed that there are less than 4% of high AOD cases with FMF lower than 0.3, which can be attributed to natural dust or oceanic coarse particles^15,17^, as well as the large ice crystals due to ice storms that frequently occur in the Arctic region^38^.

The observed aerosol microphysical parameters at the Arctic sites (see Supplementary Fig. S1) show that the high aerosol load cases (i.e., AOD at 440 nm larger than 0.4) are dominated by fine and spherical particles. The average volume concentration of coarse mode aerosol particles (in μm^3^/μm^2^), which are mostly from natural sources, is only 1/5 that of fine mode. In addition, the coarse-to-fine mode ratio is even smaller at TIK and RES (approximately 1/10), as the two sites are greatly affected by transported biomass burning particles. Spectral IRI over the Arctic (0.013 and 0.011 at 440 and 870 nm, respectively) are comparable to the urban/industrial and biomass burning aerosol^39^, likely illustrating the major influences of these aerosol types. A previous study argued that the light absorption of Arctic aerosol was mainly due to biomass burning sources^4^, which agrees well with our observation that the IRI at RES and TIK sites are much larger and the spectral dependency (i.e., higher at 440 nm than other wavelengths) is more obvious than at other sites.

### Aerosol chemical properties of high aerosol load cases over the Arctic

**Figure 3:**
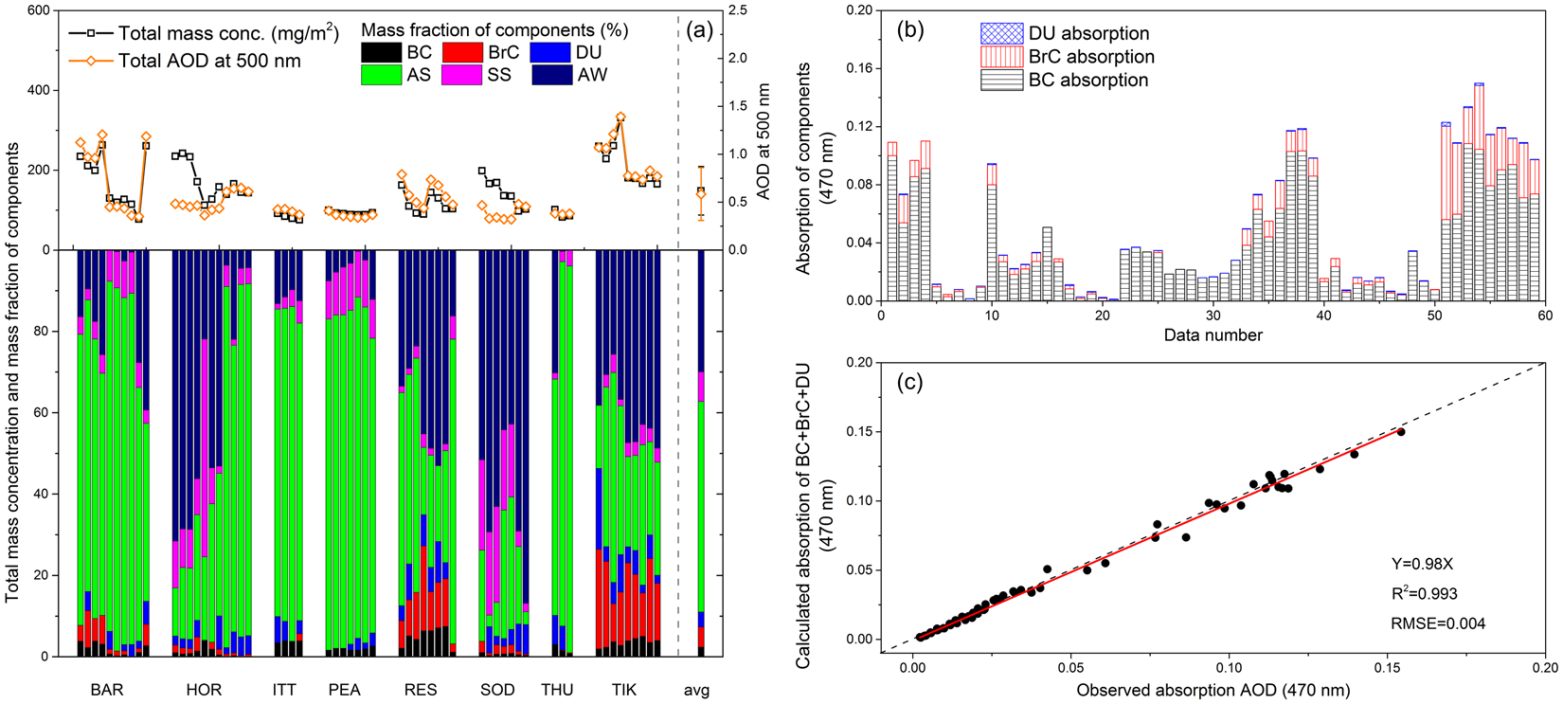
(**a**) Estimated columnar mass concentration of components at Arctic sites: total mass concentration (mg/m^2^) and AOD at 500 nm (top panel) and component mass fractions (%) (bottom panel). The average (avg) total mass concentration and component fractions of all data are separated by the dashed grey line. (**b**) BC, BrC and DU absorption estimated by mass concentrations. (**c**) Comparison between calculated total absorption and observed absorption AOD from the AERONET.

The estimated columnar total mass concentration (TMC, mg/m^2^) and component mass fractions (%) are shown in Fig. 3(a). The average TMC over the Arctic is 149 ± 61 mg/m^2^. Except for the events on May 2, 2006, at HOR and on August 4–6, 2014, at SOD, other TMC values have a good correlation with AOD (the correlation coefficient R is 0.96). Concerning these exceptional cases, we calculate the volume extinction efficiency (the ratio of AOD to volume) of fine mode aerosol using AOD, FMF and volume size distribution. We find these exceptions have small efficiencies (~3.7, only half that of other events). A possible explanation is the high content of the less efficient component (i.e., AW contents of the cases are 3 times higher than the others).

The average AS mass concentration is 70 mg/m^2^, which is approximately 68% of the total dry mass, similar to other studies^40^. This implies that the light-scattering and fine-mode inorganic salts and organic matter mainly from anthropogenic, combustion and volcanic sources dominate columnar mass under high AOD conditions in the Arctic. In comparison, BC and BrC have much lower contributions (no more than 14%), except for those at RES and TIK, where the absorbing components can reach a level comparable to that of AS. The average BrC-to-BC mass ratio of the Arctic sites is approximately 2.2, which agrees well with modelling results^9^. Compared to the fine particles, the mass concentrations of coarse-mode components are much lower^41^. The average contributions to aerosol mass of DU and SS are only 4% and 7%, respectively, confirming that high-AOD events should be mostly attributed to fine particles transported from mid-latitude regions.

As shown in Fig. 3(b), we use the mass absorption efficiency (MAE) at 470 nm of BC, BrC and DU^42^ to calculate the light absorption of each component according to the inferred columnar mass. BC, BrC and DU contribute 83%, 15% and 2%, respectively, to the total aerosol absorption, similar to modelling results^10^. We use the absorption AOD (AAOD) data from AERONET to validate the absorbing component estimates^43^ given that the light absorption computed from the MAE and the inferred composition is independent of the AAOD. As shown by Fig. 3(c), the total light absorption of the three components are consistent with the AAOD. Moreover, we conduct closure tests by simulating the microphysical parameters according to the inferred composition, and compare them with the input parameters to check whether the estimates are effective and stable. As expected, we get highly consistent results (R^2^ = 0.99, slope = 0.95–1.0; see Supplementary Fig. S2).

**Figure 4:**
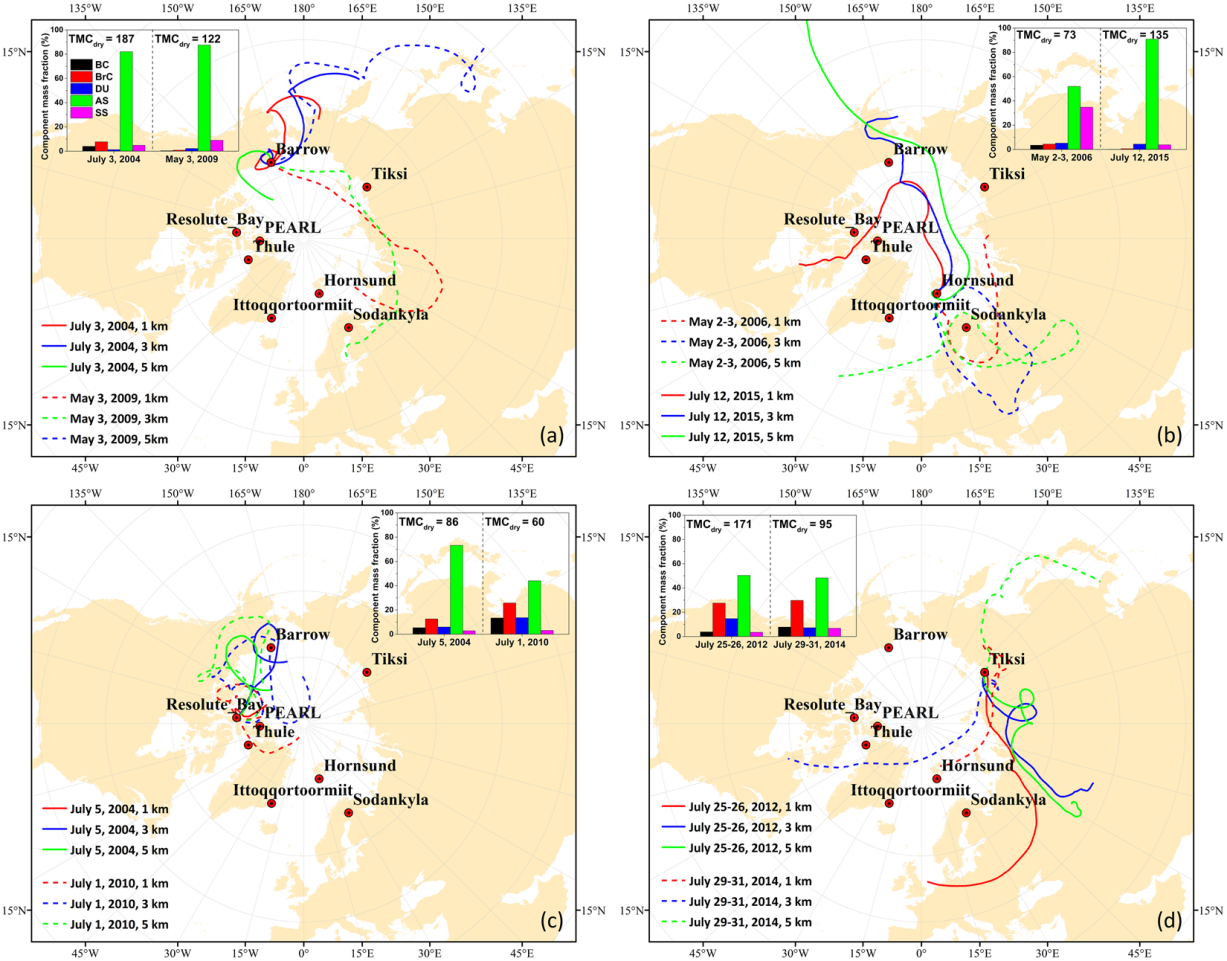
HYSPLIT backward trajectories of 10-day duration for different aerosol events at the (**a**) BAR, (**b**) HOR, (**c**) RES and (**d**) TIK. The colors indicate airmasses at different heights. The average total dry mass concentration (TMC_dry_) (unit: mg/m^2^) and component mass fractions for each event are also shown. The maps are generated using ArcGIS 10.2 software (www.esri.com/software/arcgis).

To identify the major sources of several typical aerosol events in the Arctic, the NOAA Hybrid Single-Particle Lagrangian Integrated Trajectory^44^ (HYSPLIT), forced by GADS (1 degree) and NARR (32 km) meteorology data (depending on data availability), is employed. Aerosol events for different types of sources may have distinct compositions even at the same site. Figure 4 shows the modelled backward trajectories for a 10-day duration at different heights (1 km, 3 km and 5 km) for separate events at the same site. On July 3, 2004, at BAR, the sum of BC and BrC mass is 22 mg/m^2^ (12% of the dry aerosol mass), and AS is 153 mg/m^2^ (82%), indicating boreal forest burning in Alaska and Canada^32^, which can be seen from the back trajectories. In contrast, on May 3, 2009, at the same site, BC and BrC contents are much less (2 mg/m^2^), while AS still maintains a high level (107 mg/m^2^, approximately 88% of dry mass). Combined with the associated back trajectories, the composition likely reflects a typical European pollution transport event, in which fine particles such as inorganic salts are dominant^45^. Aerosol compositions and back trajectories of the two events at HOR also imply different sources. The high BC and BrC concentrations on May 2–3, 2006, indicate major agricultural combustion sources from eastern Europe^46^. The one on July 12, 2015, in comparison, is likely caused by pollution from North America, considering the extremely low BC and BrC fraction (1% of dry mass) but very high AS content (91%).

For the two events at RES, the high contents of carbonaceous components and the back trajectories imply primary biomass burning sources from North America. Both of the events at the TIK site also show very high BC and BrC mass fraction (30–40%), which is consistent with other studies^43^. Specifically, the BrC-to-BC ratio of the event on July 25–26, 2012 reaches ~8, indicating a typical biomass burning aerosol event originating from Russia^47^. According to the aforementioned events, one notices that the biomass burning particles transported to the Arctic usually contain high BC and BrC contents (approximately 10–40% of dry aerosol mass). However, for Arctic haze or pollution events, the AS mass is extremely high (close to or exceeding 90%), while the light-absorbing components occupy only approximately 1% of the dry aerosol mass.

### Aerosol radiative properties of high aerosol load cases over the Arctic

**Figure 5:**
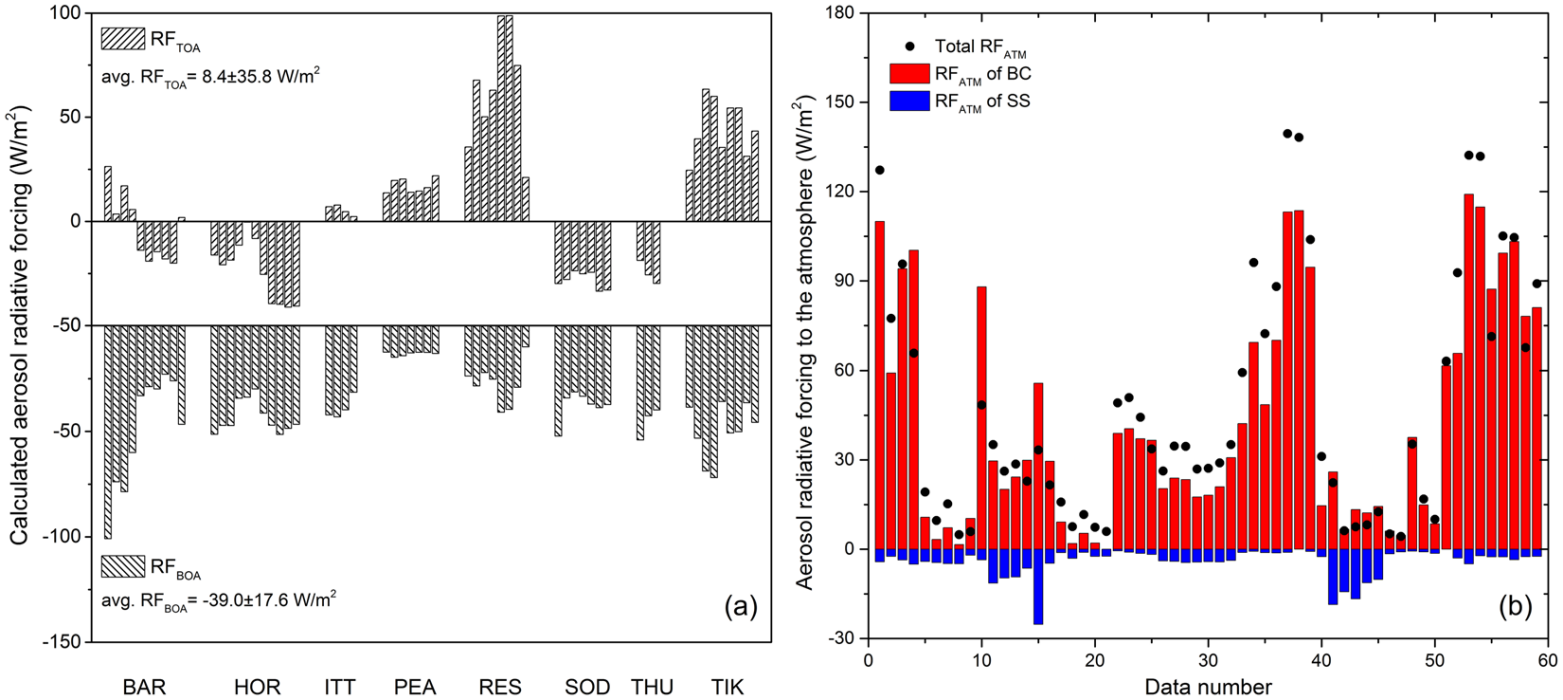
(**a**) Calculated RF_TOA_ (upper bar chart) and RF_BOA_ (lower bar chart) of Arctic sites. (**b**) RF_ATM_ for total aerosol (black dots) and for the BC and SS components (color bars) according to the regression model.

The RF_TOA_, RF_BOA_ and RF_ATM_ of the high aerosol load cases over the Arctic are shown in Fig. 5. The average RF_TOA_ is approximately 8.4 W/m^2^, with remarkable differences among sites (ranging from −41.2 to 98.7 W/m^2^). The RF_TOA_ generally increase with surface albedo for small to medium albedo values; when albedo >0.5, most RF_TOA_ values are positive, and vice versa^48^ (see the Supplementary Fig. S3). This tendency is due to the upward fluxes at TOA from surface reflection increasing faster as albedo increases under clear conditions than under the presence of aerosol. The influence of solar zenith angle (SZA) on RF_TOA_ is investigated under different surface albedos (see Supplementary Fig. S4). We find that for bright surfaces (albedo >0.5) the positive RF_TOA_ decreases as SZA increases, consistent with previous studies^48^. This is because the upward fluxes at the TOA without aerosol decrease more rapidly with SZA than those with aerosol. For dark surfaces (albedo less than 0.2), the behavior is not obvious (see Supplementary Fig. S4). The estimated BC columnar mass concentration correlates with the RF_TOA_ (R = 0.82), and most RF_TOA_ values are positive when BC exceeds 3 mg/m^2^ (see the Supplementary Fig. S5), because the absorbing component reduces radiation backscattered towards the TOA^29^. The rate of increase of RF_TOA_ with surface albedo is higher for large BC content because the upward fluxes at the TOA increase more slowly with surface albedo for strongly absorbing aerosols than weekly absorbing aerosols.

The RF_BOA_ values over the Arctic are mostly negative, with an average of −39.0 ± 17.6 W/m^2^, i.e., cooling at the surface^48^. The cooling is enhanced when aerosol mass is high, as expected, especially for the absorbing components^29^. The calculated RF_ATM_ values are positive, with an average of 47.3 ± 39.9 W/m^2^ (see Fig. 5b), implying warming in the atmospheric column over the Arctic^45^. RF_ATM_ varies considerably even at the same site. For example, the RF_ATM_ at BAR ranges from over 120 W/m^2^ to less than 10 W/m^2^, with little change in surface albedo and solar zenith angle. This is probably due to variation in the absorbing component content since high BC and BrC mass can result in a large, positive RF_TOA_ and a large, negative RF_BOA_. This reflects the important contributions of absorbing components on atmospheric heating. It should be noted that the RF values analysed above are not representative of the average conditions but rather indicate only the aerosol radiative properties of relatively infrequent high-AOD events, and for most of the clean conditions in the Arctic region, aerosol impacts on radiation are expected to be relatively small.

To analyze the relation between component mass concentrations and RF_ATM_ under high-AOD conditions we use a multiple linear regression model (SPSS, stepwise-method):

5 $$R{F}_{ATM}=c+\sum _{i=1}^{n}{a}_{i}\times C{M}_{i}$$

where *CM*_*i*_ is the columnar mass concentration of the *i*th component. The coefficient *a*_*i*_ denotes RF_ATM_ per unit columnar mass concentration (in W/mg) of individual components, which can be interpreted as the warming (positive) or cooling (negative) efficiency of aerosol components (*a*_*BC*_ = 12.2, *a*_*SS*_ = −0.4; for other components, *a*_*i*_ equals 0). The model constant *c* indicates the model bias (*c* = 9.0). The coefficients are statistically significant according to t-test results (p values for *a*_*BC*_, *a*_*SS*_, and *c* are <0.001, 0.004 and 0.004, respectively). The adjusted determination coefficient over 0.9 and the small standard error of the estimate illustrate that this model is statistically effective.

The standardized partial regression coefficients for BC and SS are 0.93 and −0.12, respectively, indicating the relative impacts of these components on RF_ATM_. Figure 5(b) shows that the absorbing BC has a dominant warming effect on the atmosphere, with a very high warming efficiency (~12.2 W/mg). It is similar to the value calculated by the surface BC concentration, mixing layer height and BC-induced RF_ATM_ from a previous study (12.9 W/mg)^49^ (columnar mass concentration can be approximated by the surface mass concentration times the layer height, assuming a vertically uniform distribution within the mixing layer^23^). It is worth noting that AS has little impact on RF_ATM_ although its contribution to dry mass is dominant, because of its efficiency at the BOA is similar to that at the TOA (−0.3 W/mg).

## Conclusions

Columnar aerosol component mass concentrations of high aerosol load cases for 8 Arctic sites are obtained from the microphysical parameters retrieved from the AERONET archive. The uncertainties of the input parameters are propagated to the resulting aerosol composition. The total light absorption calculated from the inferred absorbing components agrees well with observed absorption AOD. The HYSPLIT back trajectory model is employed to identify the major emission sources contributing to the observed high aerosol load events over the Arctic. The estimated aerosol composition, together with aerosol optical, microphysical and radiative properties from AERONET data, show the dominance of fine mode aerosols over the Arctic, especially for high aerosol load cases.

The multiyear average AOD at 500 nm for the sampling periods and sites is 0.08 ± 0.08, with an average FMF of 0.75 ± 0.16. In particular, approximately 91% of large AODs (i.e., >0.4) correspond to very high FMFs, implying a significant contribution to total light extinction from fine particles.

For high aerosol load cases in the Arctic, columnar aerosol components, including BC, BrC, DU, AS, SS and AW, are estimated from microphysical parameters, including refractive index, size distribution and spherical fraction. The light-scattering and fine-mode component AS dominates aerosol dry mass (approximately 68%). BC and BrC make smaller contributions to aerosol mass, but account for most of the total aerosol light absorption. DU and SS components that are mainly from natural sources have much lower mass concentrations compared to the fine components.

According to the HYSPLIT back trajectories of the high aerosol events in the Arctic, events originating from biomass burning usually correspond to high BC and BrC mass fractions (10–40%), but for pollution events, AS fractions are extremely high (approximately 90%), while the fractions of light-absorbing components are small.

The considerable variations in RF_TOA_ over the Arctic are probably due to varying BC mass, as well as surface albedo and solar zenith angle. The average RF_ATM_ for high aerosol events is 47.3 ± 39.9 W/m^2^, indicating significant aerosol warming effects on the atmosphere under high-AOD conditions. The composition-RF_ATM_ regression model shows that BC contributes most to the RF_ATM_, with a high warming efficiency (12.2 W/mg), while SS has a minor cooling effect.

## Electronic supplementary material

Supplementary information
