# Supplementary material for: Aerosol optical, microphysical, chemical and radiative properties of high aerosol load cases over the Arctic based on AERONET measurements
Source: Sci Rep. 2018 Jun 20;8:9376. doi: 10.1038/s41598-018-27744-z (PMC6010420; doi:10.1038/s41598-018-27744-z)
Supplement: Supplementary file 1 — Supplementary information [file 41598_2018_27744_MOESM1_ESM.pdf]

Supplementary information

Aerosol optical, microphysical, chemical and radiative properties of high aerosol load cases over the Arctic based on AERONET measurements

Yisong Xie[1], Zhengqiang Li[1,*], Li Li[1], Richard Wagener[2], Ihab Abboud[3], Kaitao Li[1], Donghui Li[1], Ying Zhang[1], Xingfeng Chen[1], and Hua Xu[1]

[1]Environment Protection Key Laboratory of Satellite Remote Sensing, Institute of Remote Sensing and Digital Earth, Chinese Academy of Sciences, Beijing, 100101, China

[2]Environmental & Climate Sciences Department, Brookhaven National Laboratory, Upton, New York, 11973, USA

[3]Measurement and Analysis Research Section, Environment and Climate Change Canada, Ontario, L0L1N0, Canada

[*]lizq@radi.ac.cn

Table S1. Assessed errors of estimated volume fractions (ΔVF%) of aerosol components, which are caused by uncertainties (Un.) of AERONET parameters, including spectral average of real refractive index (RRI_{avg}), imaginary refractive index at 440 and 870 nm (IRI_{440} and IRI_{870}), fine-mode volume fraction (FVF) that calculated from size distribution, and spherical fraction (SPH). At first, add perturbations step by step to each of the parameters according to the uncertainty ranges; then infer component volume fractions according to the input parameters with and without perturbations; at last calculate the differences between the original and perturbed volume fractions as the errors. The values indicate possible error ranges of components due to individual parameter. The errors listed in the last row show the largest error of each components, and the values in parentheses are the estimated fractions without perturbation.

	**ΔVF_{BC}**	**ΔVF_{BrC}**	**ΔVF_{DU}**	**ΔVF_{AS}**	**ΔVF_{SS}**	**ΔVF_{AW}**
**Un. RRI_{avg}**	0	-0.6~+0.2	0	-23~+23	0	-22.6~+23
**Un. IRI_{440}**	-0.3~+0.1	-3~+8.4	0	-9~+4	0	-0.8~+0.6
**Un. IRI_{870}**	-0.5~+0.3	-2.8~+4.2	0	-3~+2	0	-0.8~+0.6
**Un. FVF**	0	-0.2~0	-2~0	-9~+6	-4~+11	0~0.2
**Un. SPH**	0	-1.6~0	0~+13	-6~0	-4~0	-1.4~0
**Maximum**	0.5 (1.8)	8.4 (3)	13 (3)	23 (49)	11 (4)	23 (39.2)

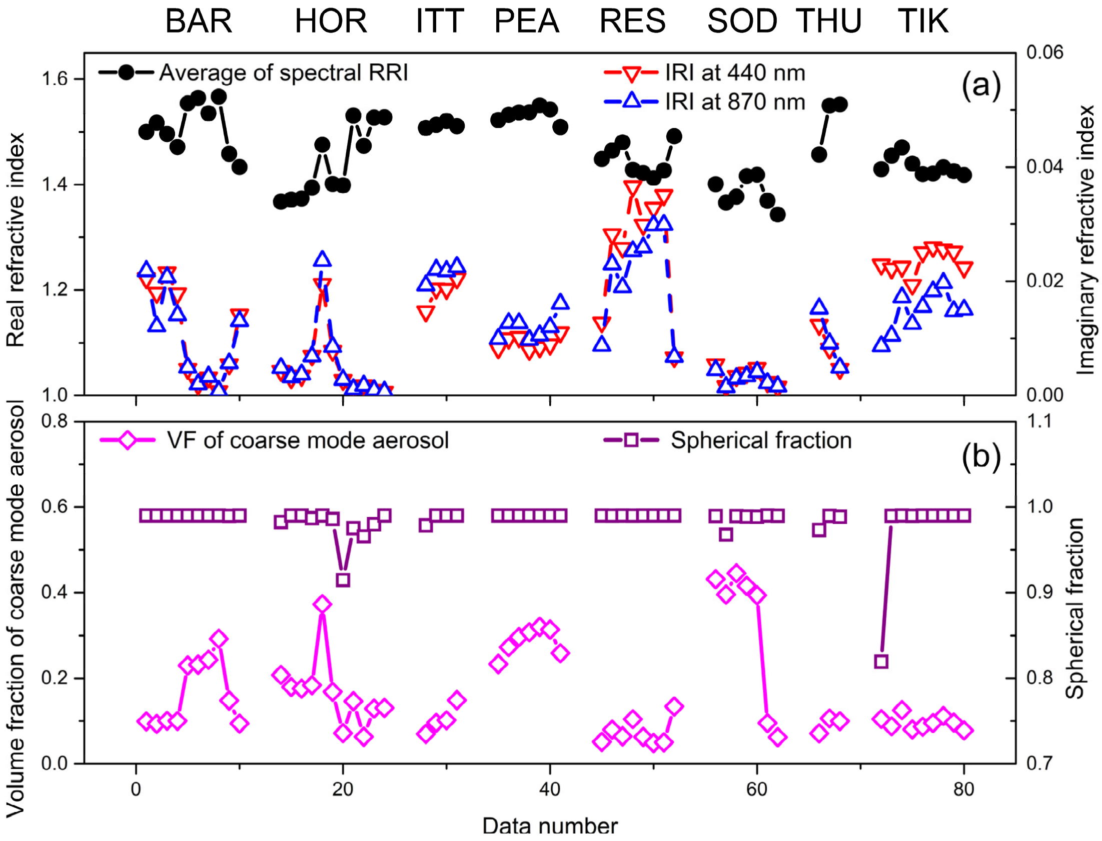

Figure S1. Aerosol microphysical properties of the Arctic sites: (a) real and imaginary refractive index, (b) volume fraction of coarse mode aerosol and spherical fraction.

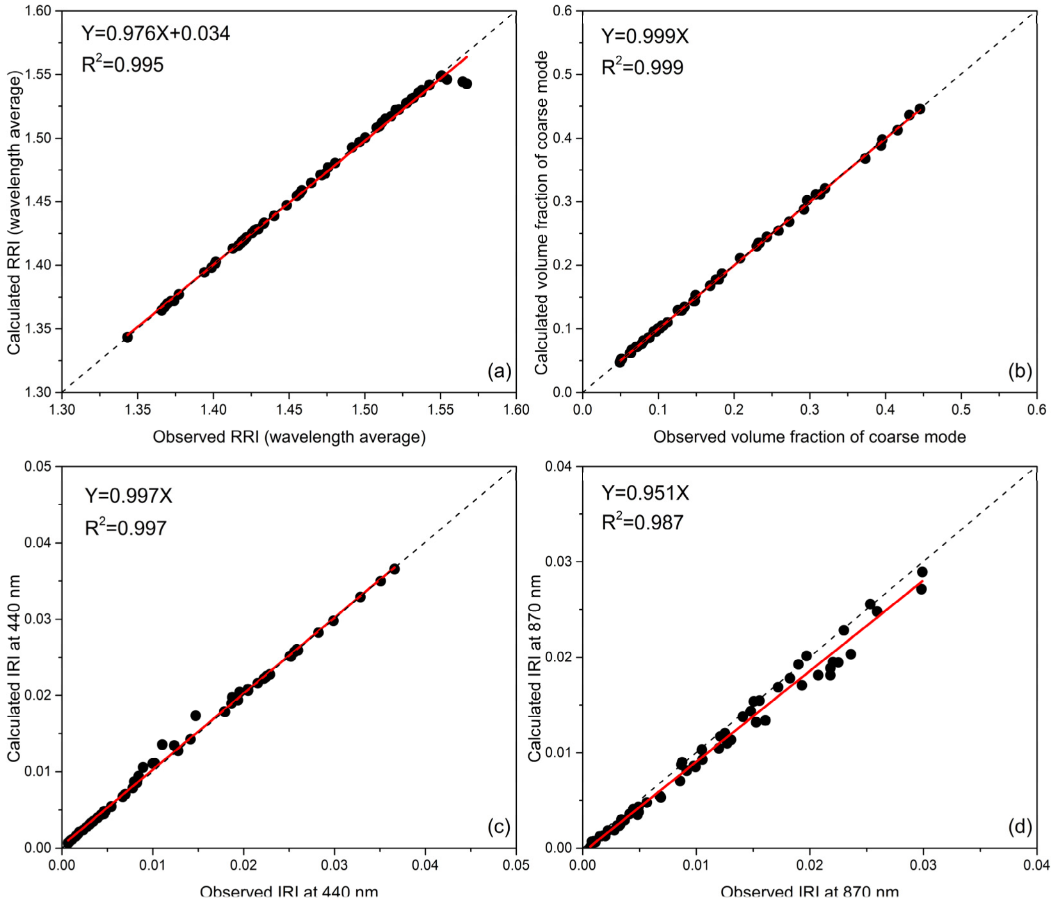

Figure S2. Comparisons of microphysical parameters between observations (AERONET products) and calculations (conducted from the retrieved components): (a) wavelength average of real refractive index (RRI), (b) volume fraction of coarse mode aerosol, (c) imaginary refractive index (IRI) at 440 nm, and (d) IRI at 870 nm.

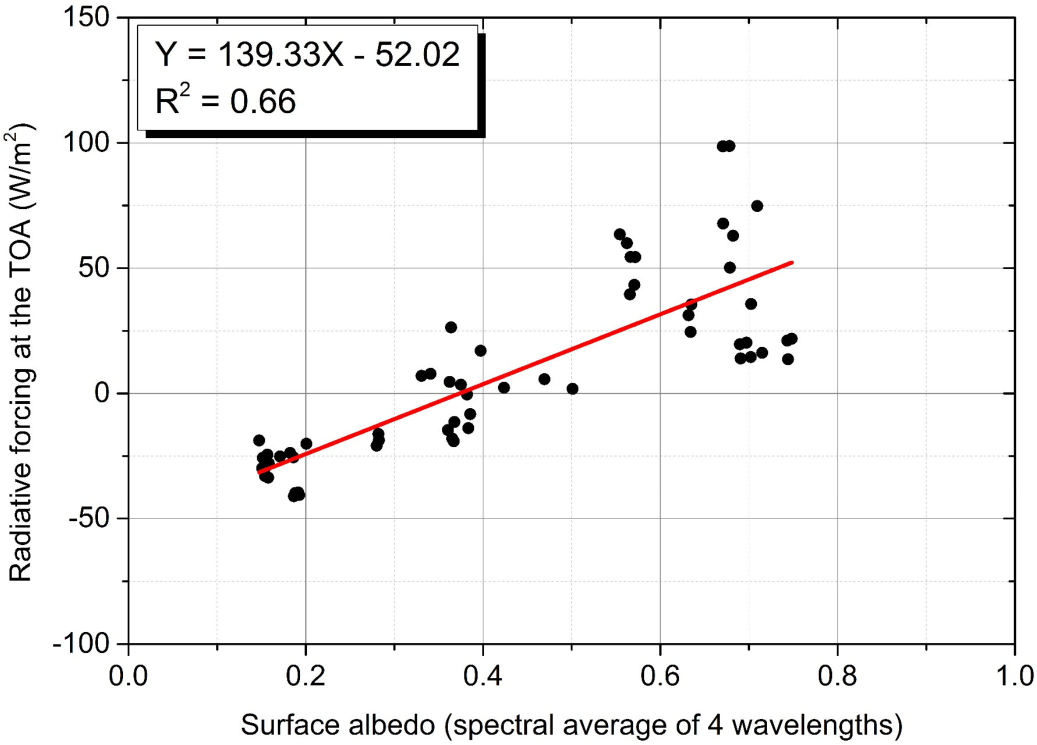

Figure S3. Aerosol radiative forcing at the top-of-atmosphere (TOA) against surface albedo over the Arctic.

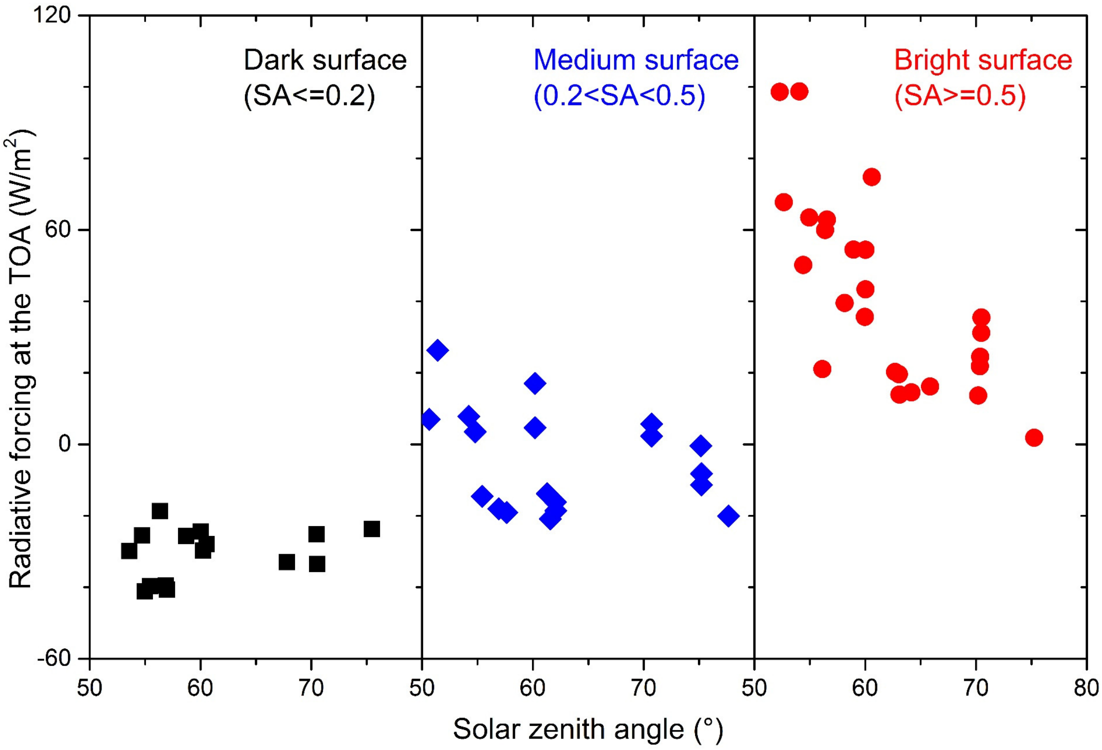

Figure S4. Aerosol radiative forcing at the top-of-atmosphere (TOA) against solar zenith angle for different sub-ranges of surface albedo (SA).

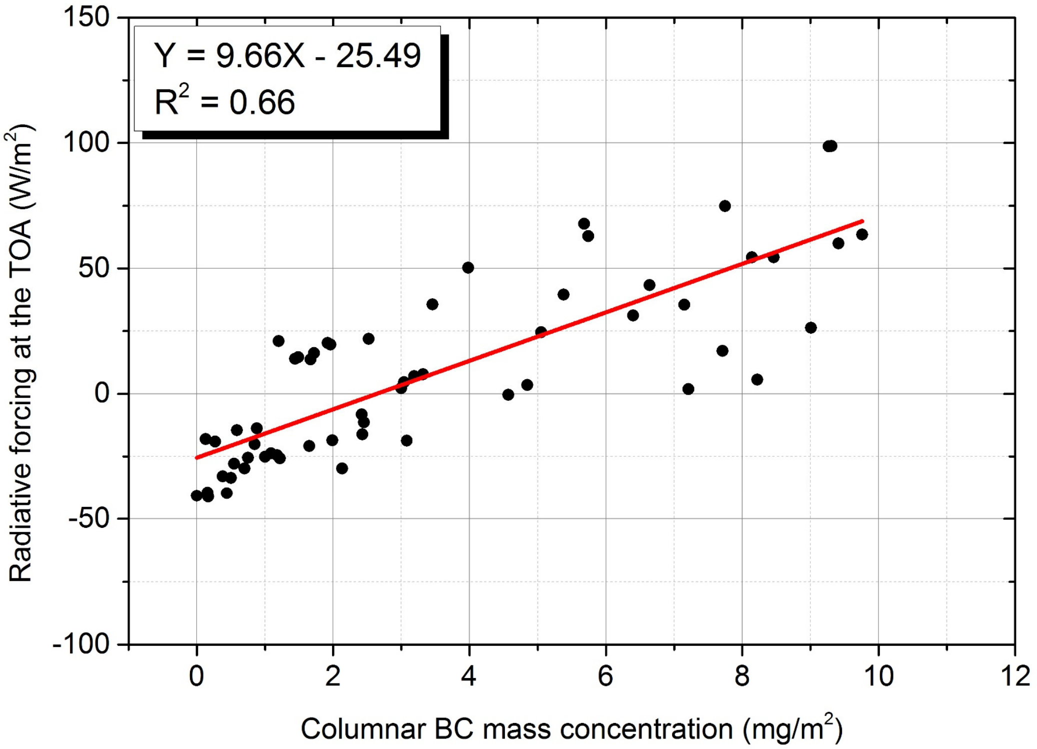

Figure S5. Aerosol radiative forcing at the top-of-atmosphere (TOA) against the estimated columnar BC mass concentration.
